# 575. PREFER-LA: Improved Adherence and Viral Control in Real-World Study of People with HIV (PWH) in the United States with Adherence Challenges on Oral Antiretroviral Therapy (ART) Switching to Cabotegravir + Rilpivirine Long-Acting (CAB+RPV LA)

**DOI:** 10.1093/ofid/ofaf695.184

**Published:** 2026-01-11

**Authors:** William R Short, Rebecca Glassman, Christina Harbison, Mitchell Whitehead, Katie L Mycock, Neil Reynolds, Hannah Wallis, Mona Amet, Ann Linskey, Jimena Patarroyo, Deanna Merrill, Edgar T Overton, Cindy Garris, Andrew P Brogan

**Affiliations:** University of Pennsylvania, Philadelphia, PA; Westchester Medical Center Health Network, Hawthorne, New York; Prism Health North Texas, Dallas, Texas; AIDS Healthcare Foundation, Pensacola, Florida; Adelphi Real World, Bollington, England, United Kingdom; Adelphi Real World, Bollington, England, United Kingdom; Adelphi Real World, Bollington, England, United Kingdom; Adelphi Real World, Bollington, England, United Kingdom; ViiV Healthcare, Durham, North Carolina; ViiV Healthcare, Durham, North Carolina; ViiV Healthcare, Durham, North Carolina; ViiV Healthcare, Durham, North Carolina; ViiV Healthcare, Durham, North Carolina; ViiV Healthcare, Durham, North Carolina

## Abstract

**Background:**

Cabotegravir + rilpivirine long acting (CAB +RPV LA) has demonstrated superior efficacy when compared to oral therapy in a randomized controlled trial of people with HIV (PWH) with adherence challenges (LATITUDE). Real-world adherence and clinical outcomes from PREFER-LA (Perspectives on Treatment with CAB+RPV LA Injectable Therapy from PWH in the US with Prior Adherence Challenges to Oral ART) are presented.

**Table 1.**
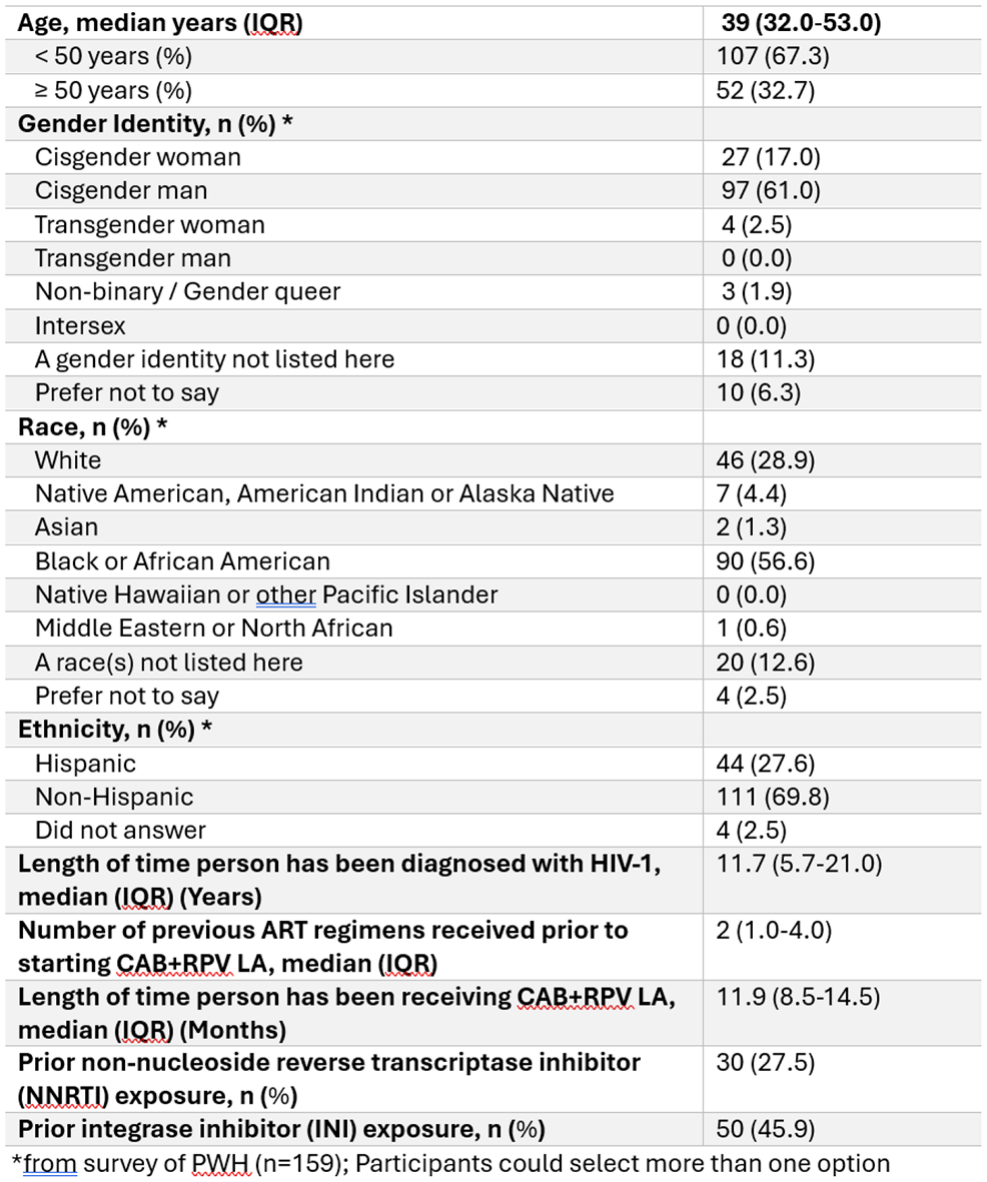
PWH characteristics and demographics (eCRF, n=159)

**Figure 1.**
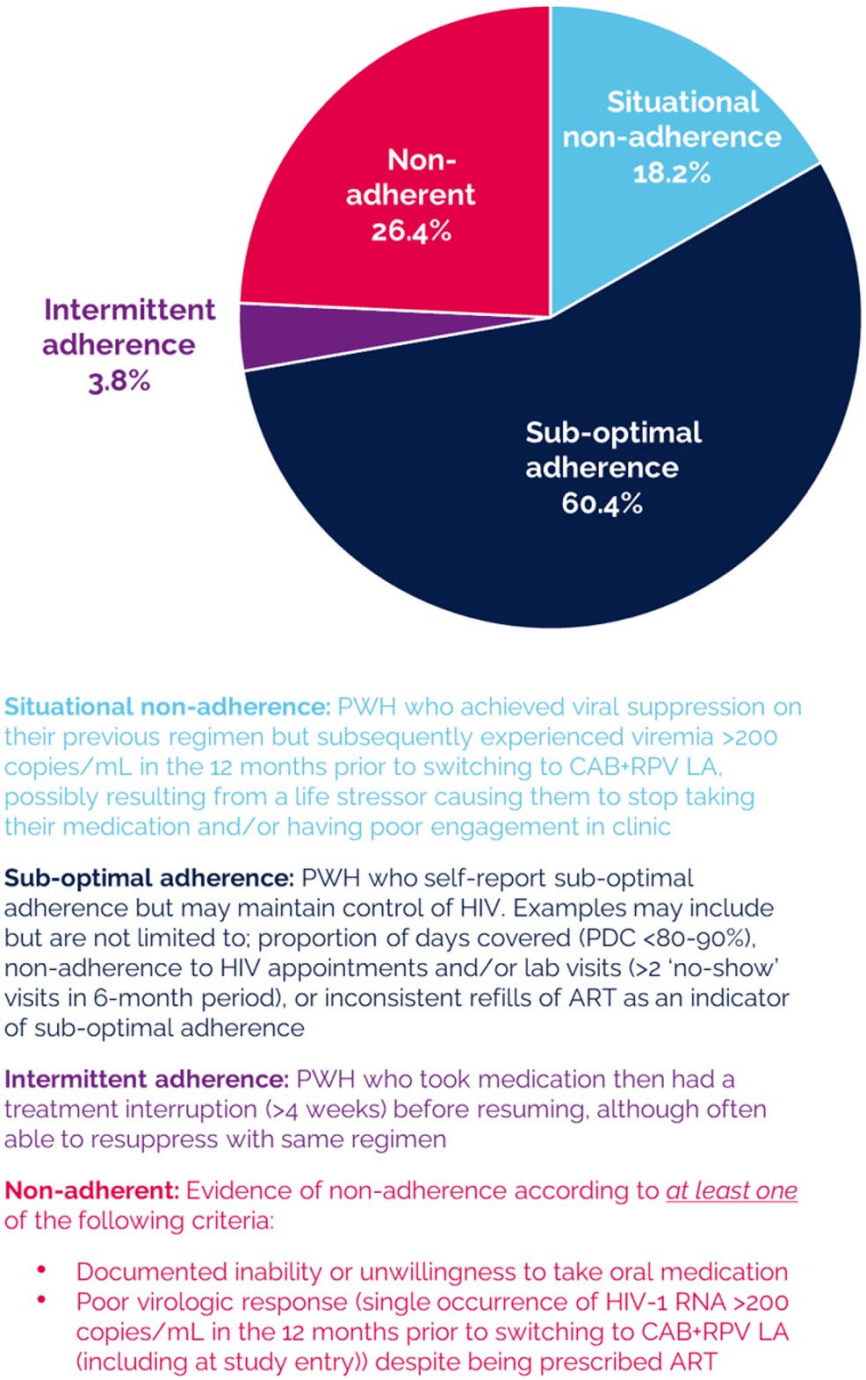
HCP reported type adherence challenge PWH experienced when receiving their previous oral ART (eCRF, n = 159)

**Methods:**

PREFER-LA was an observational real-world US study of PWH receiving CAB+RPV LA for ≥6 months to ≤18 months with documented adherence challenges on prior oral ART. The study consisted of: (1) cross-sectional survey of PWH to evaluate experiences of historical oral ART use and perspectives of treatment with CAB+RPV LA, (2) corresponding retrospective medical chart review (eCRF) to establish treatment history and clinical outcomes, and (3) cross-sectional survey of healthcare providers (HCP) from each site.

**Figure 2.**
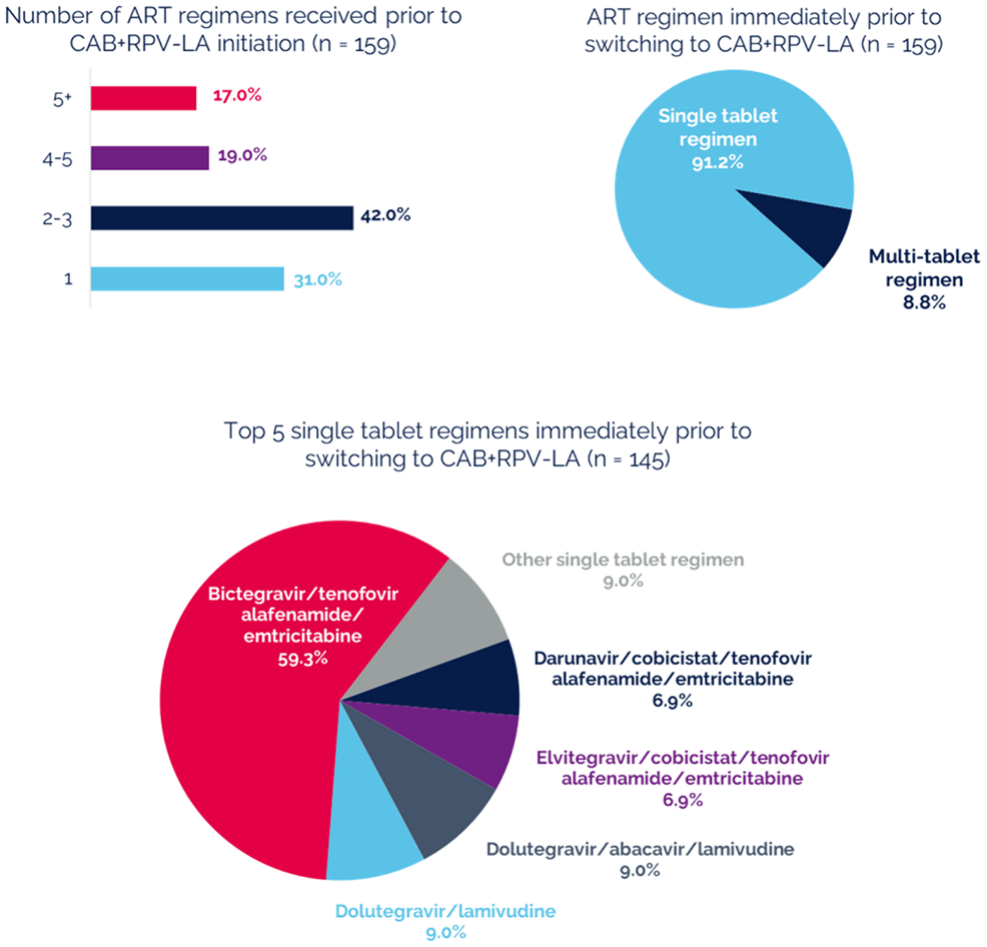
PWH prior ART history (eCRF)

**Figure 3.**
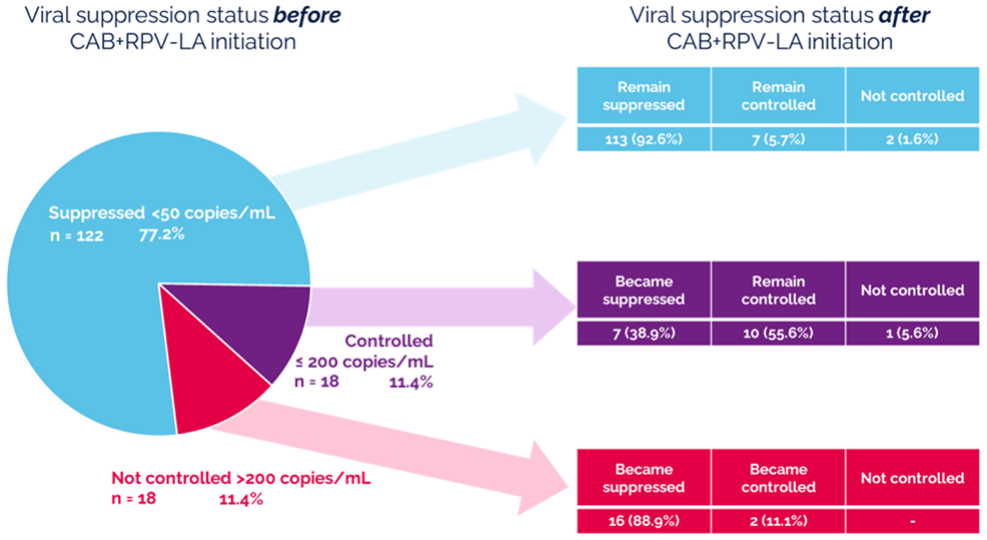
Viral suppression status before and after switch to CAB+RPV LA (eCRF, n = 159)

**Results:**

Median age of the 159 participants was 39 years and median time since HIV diagnosis was 11.7 years (Table 1). HCPs identified the type of sub-optimal adherence for each PWH for study eligibility (Figure 1). HCPs reported forgetfulness (64%) as the most common factor impacting daily oral ART adherence. From the PWH perspective, remembering to take oral ART was the most common adherence challenge (84%). For nearly 70% of PWH, CAB+RPV LA was at least their third ART regimen (Figure 2). Over 90% of PWH switched from a single tablet oral ART; bictegravir/tenofovir alafenamide/emtricitabine (59%) was the most common prior single tablet oral ART. No resistance data were available at time of switch for 38% of PWH. Nearly one quarter of PWH had a viral load >50 copies/mL at time of switch (Figure 3). At the time of evaluation, median follow-up time on CAB+RPV LA was 1 year, and viral load < 200 copies/mL was achieved/maintained in over 98% of PWH (Figure 3). Missed/skipped/delayed CAB+RPV LA injections were uncommon (13%).

**Conclusion:**

In this cohort of PWH with documented adherence challenges to oral ART, virological control was achieved/maintained by most after switching to CAB+RPV LA. These real-world findings complement existing clinical trial and real-world evidence regarding use of CAB+RPV LA in PWH with identified adherence challenges on prior oral ART.

**Disclosures:**

All Authors: No reported disclosures

